# On-Line Foreign Object Detection Using Double DD Coils in an Inductive Wireless Power Transfer System

**DOI:** 10.3390/s22041637

**Published:** 2022-02-19

**Authors:** Nataša Prosen, Miro Milanovič, Jure Domajnko

**Affiliations:** Faculty of Electrical Engineering and Computer Science, University of Maribor, 2000 Maribor, Slovenia; miro.milanovic@um.si (M.M.); jure.domajnko2@um.si (J.D.)

**Keywords:** WPT, double DD coil structure, mutual inductance measurement, coupling coefficient measurement, FOD, on-line measurement

## Abstract

This paper proposes an on-line method for foreign object detection in a double DD coil system. The foreign object is detected by real-time measurement of the mutual inductance between the transfer pads. Measurement of the mutual inductance between coils can be performed at the start, during initialisation, or during the wireless power transfer. The coils in the double DD coil structure can be used separately; one coil can be used for power transfer and the other can be used for the mutual inductance measurement. The mutual inductance measurement is based on the voltage measurement across the open circuit receiver coil. The measured value of mutual inductance between the transmitter and the receiver pad can be used in a control algorithm and in a foreign object detection algorithm. Additionally, a 2DDq coil structure can be used as a replacement for the double DD coil structure, which increases the power transfer density. The DD coils in the double DD coil structure can also be driven using two phase-shifted voltages, which enables better location and detection of foreign objects. The method also helps to differentiate the mutual inductance change due to the distance change from the mutual inductance change due to the presence of a foreign object.

## 1. Introduction

Research in the field of wireless power transfer (WPT) received more interest due to the rapid development of the mobile and automotive fields [1,2,3,4]. The conventional wireless transfer system transfers power between a transmitter and the receiver side, which can be separated by a distance that can range from a couple of millimetres to a couple of metres. Power transferred using a contactless WTP system can vary from a mW to a couple of kW, depending on the application. There are different wireless transfer technologies—capacitive wireless transfer (CPT), inductive wireless power transfer (IPT), and wireless transfer using a laser and a photovoltaic panel [5]. The most popular of these is IPT, which is usually used in medium ranges, from a couple of centimetres to a couple of decimetres, and enables power transfer up to several kWs [6]. The main advantages of WPT are its robustness and ease of use compared to conventional wired power transfer. However, the wireless power transfer also introduces several new problems, particularly its lower and position dependent efficiency and increased system complexity.

In IPT, the efficiency of the system is highly dependent on mutual inductance between the transmitter and the receiver coil. This can be expressed as the coupling coefficient *k* between the transmitter and the receiver coil. In a typical application, the coupling coefficient is smaller than 0.5. The inductive resonant method can be used to increase the efficiency of a wireless power transfer [7]. Even though the efficiency of the system is increased, it is still highly dependent on misalignment between the receiver and the transmitter coil.

The misalignment and the distance between the transmitter and the receiver coil can be estimated using the measurement of the mutual inductance between the transmitter and receiver. The mutual inductance can also be used to optimise control of the IPT system and can be measured using several different methods. The simplest method is measuring the series-connected transmitter and receiver circuit with an LRC meter, and then calculating the mutual inductance from the measured inductance [8]. It can also be calculated using the measurement of the open-loop-inducted voltage on either the primary or secondary side. These methods cannot be used during wireless power transfer. It is possible to estimate mutual inductance using measurements of voltages and currents in primary and secondary circuits [9,10,11,12,13,14,15]. Estimations are based on simple circuit equations that describe the compensated resonant IPT. On-line mutual inductance can be estimated from primary and secondary parameter measurements, or simply from a primary circuit measurement. The authors of [16] propose estimation using Fourier analysis of the primary voltages and currents. The on-line mutual inductance measurement can be used to detect foreign objects located in the magnetic field, in addition to control optimisation of the IPT system.

Foreign objects are metallic objects that can appear between the transmitter and the receiver pads. The magnetic field induces voltage in the object; consequently, the object can become hot, especially in a high-power wireless power transfer. Foreign objects can include cans, coins, keys, and larger metallic objects. Due to the heat generated by the induced eddy currents, foreign objects can injure humans and animals. Foreign objects also lower system efficiency because they increase IPT system losses. The methods for detecting foreign objects in the magnetic field are called foreign object detection (FOD) methods. A review of FOD detection methods is presented in [11].

The IPT system described in this paper uses double DD coils on both the primary and secondary sides [17]. This enables simultaneous wireless power transfer and mutual inductance measurement. The transferred power is halved during measurement. The value of the mutual inductance can be used for control optimization or for foreign object detection, which is of particular interest (FOD). An additional coil structure is presented in order to increase the transferred power. The proposed coil structure consists of three coils. Two coils are used to transfer power, and one coil is used to measure the mutual inductance between the transmitter and the receiver coil.

The proposed FOD method is used at the start of the wireless power transfer process. Therefore, the control of the IPT system during the power transfer is not included in this paper.

The paper is organised as follows. After the Introduction, the measurement method using the double DD coils is described in Section 2. Section 3 describes the mathematical model of the IPT system using the double DD coil and measurement system. The extension of the system in order to increase the power transfer density is presented in Section 4. The use of the measured mutual inductance in the FOD and the FOD algorithm are described in Section 5. Next, Section 6 describes the IPT experimental set-bench. The results, measured using the experimental set-bench, are presented and evaluated in Section 7. Section 8 is a discussion about the experimental results and limitations. Finally, Section 9 serves as the conclusion of this paper.

## 2. Method Description

The wireless power transfer system described in this paper consists of a transmitter and a receiver with a special coil structure, called a double DD coil structure. DD coils are named after their shape, and were first presented in [18,19]. The DD coil shape consists of two planar spiral coils called D coils. The difference between a standard planar spiral square or circular coil and a DD coil is that the DD coil generates uniform directed flux, similar to a flux-pipe. The advantages of uniform flux are its better coupling coefficient and better misalignment tolerance.

**Figure 1:**
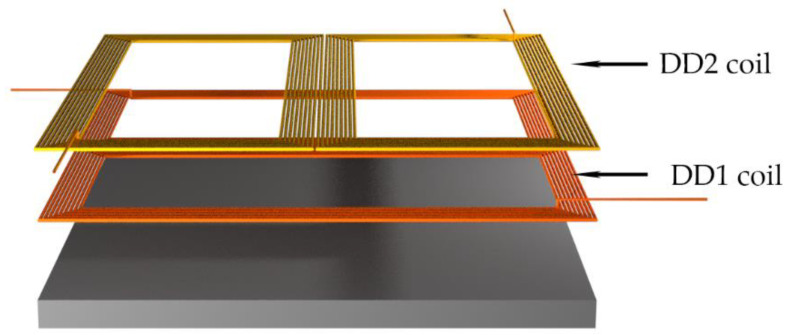
Double DD transfer pad.

Compared to a classic single DD coil pad structure, the double DD coil pad structure implements two DD coils with the same parameters, which are rotated to each other by 90 degrees [17]. The double DD structure is presented in Figure 1. The pad structure consists of a DD1 coil on the bottom, a DD2 coil on the top, and a shielding ferromagnetic plate. The DD2 coil is placed on top of the DD1 coil. Due to a directional and perpendicular magnetic field, the DD1 and DD2 coils are magnetically uncoupled. In the optimal position, the DD1 transmitter coil generates a magnetic field, which induces voltage only in the DD1 receiver coil. Similarly, the DD2 transmitter generates a magnetic field, which induces voltage only in the DD2 receiver coil. The generated fields are perpendicular to one another.

The double DD coil structure therefore enables wireless power transfer using two separated channels. Both channels can be used for wireless power transfer; therefore, the system can be used as a wireless power transfer system using two transmitters and two receivers.

In an on-line measurement of the mutual inductance, one pair of DD coils, or channel, can be used for power transfer. The second channel, or other pair of coils, can be used for mutual inductance measurement. The first and second channels do not interact with each other. To measure the mutual inductance between the transmission pads, one coil can be used in normal mode to transfer power, and the other one can be used to measure the value of the mutual inductance between the pads, using the measurement of voltage induced on the secondary, receiver side. Equations that describe the induced voltage are presented in Section 3, under the IPT system circuit analysis. For instance, the DD1 coil can be used for wireless power transfer, and the DD2 coil can be used for mutual inductance measurement. The value of mutual inductance can be used for control optimization, load detection, and foreign object detection.

**Figure 2:**
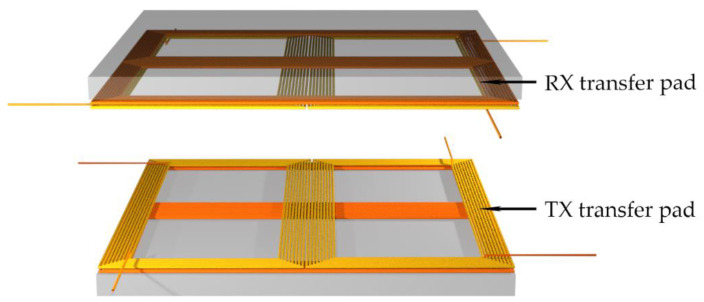
Position of the double DD transfer pads in a 3D space.

For the proposed coil structure to interact and work correctly, the TX and RX coils must be rotated to each other correctly. The perfectly aligned position of the pads is presented in Figure 2. The transmitter DD1 coil induces voltage in the receiver DD1 coil and the transmitter DD2 coil induces voltage in the DD2 receiver coil. If the transmission pads are rotated to each other, the DD1 transmitter coil induces voltage in both the DD1 and DD2 receiver coils, which couples a DD1 and DD2 resonant circuit.

To generate a current and magnetic field in both wireless transfer channels, the transmitter coil must be connected to a high-frequency inverter that can drive the two separated transmitter coils. The simplest way, which was also used in the case of this paper, is to drive the double DD coil using a single full-bridge inverter. The compensation circuits of both DD1 and DD2 coils were connected between one of the two inverter full-bridge legs. The inverter generated a positive square driving voltage. The transmitter DD1 and DD2 coils resonate with their respective compensation networks, forming a band-pass filter that filters the square driving voltage. If the driving voltage frequency and band-pass filter resonant frequency are the same, the resonator behaves as a pure resistive load. A sinusoidal current that generates a magnetic field on both channels runs through each coil. The two full-bridge inverters can also generate two driving currents that each have a phase difference, which is crucial in generating the rotating directional magnetic field, described further in Section 4.

On the secondary side, the magnetic field of one channel can be used together with the receiver, compensation circuit, and rectifier to transfer power to the resistive load. The other channel can be used for mutual inductance and coupling coefficient measurement. When the measurement is completed, the second channel can also be connected to the compensation circuit, rectifier, and load. The mutual inductance measurement can be performed at the start of or during the wireless power transfer. If measurement is performed during the wireless power transfer, the transferred power is reduced by half, due to only one channel being used for the power transfer.

## 3. Mutual Inductance Measurement Using Double DD Coils

### 3.1. The IPT System Circuit Analysis

**Figure 3:**
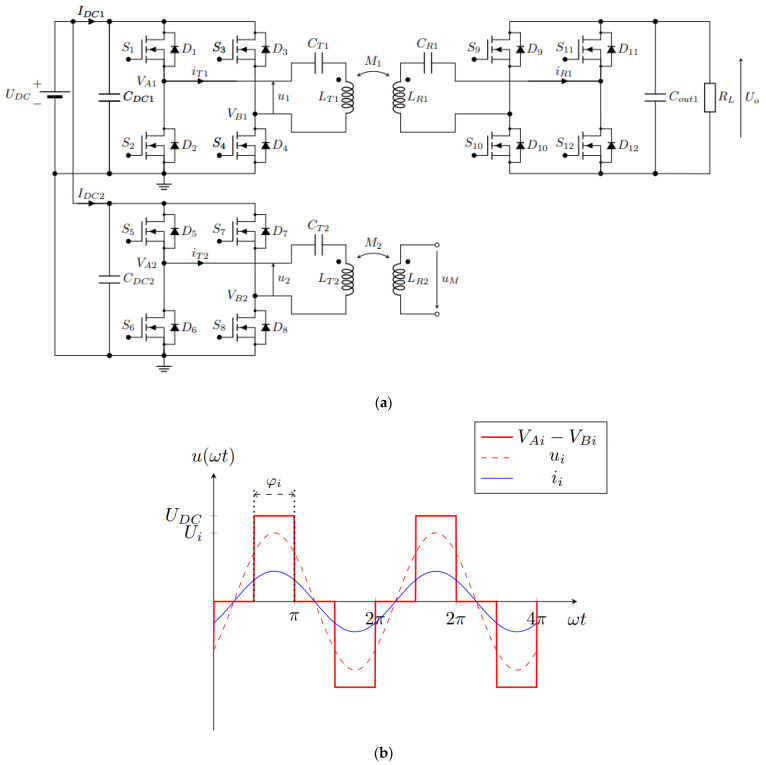
The system structure of IPT using a double DD coil structure. (**a**) The system structure; (**b**) The voltage and current waveforms at the input of the resonant circuit.

The basic circuit of the system is presented in Figure 3a. It consists of two full-bridge high-frequency inverters that drive two series-compensated (S) transmitter DD coils with currents *i_T_*_1_ and *i_T_*_2_. The DD1 transmitter coil with self-inductance *L_T_*_1_ is coupled magnetically with the receiver DD1 coil with the self-inductance *L_R_*_1_. The DD2 transmitter coil with inductance *L_T_*_2_ is coupled magnetically with the DD2 receiver coil with inductance *L_R_*_2_. The DD1 coils are used to transfer power from the transmitter to the receiver side and power up the load *R_L_*. The DD2 coils are used to measure and calculate the mutual inductance between the coils using the open circuit voltage measurement method.

If there is no misalignment between the transmitter and the receiver pad, the coupling coefficients between the two DD1 coils and two DD2 coils are the same. This means that the measurement of the mutual inductance between coils *L_T_*_2_ and *L_R_*_2_ is the same as the mutual inductance between coils *L_T_*_1_ and *L_R_*_1_, which are used to transfer the wireless power. This enables online mutual inductance measurement. When mutual inductance is measured, the *L_R_*_2_ receiver coil can be connected back to the rectifier and load.

The transmitter and the receiver pad are compensated using series–series compensation topology (SS), using capacitors *C_T_*_1_, *C_T_*_2_, and *C_R_*_1_. These components form a DD1 resonator circuit that transmits power to the load. In the case of voltage measurement, the receiver DD2 coil with inductance *L_R_*_2_ does not require a compensation capacitor. When the measurement is finished, the coil can be connected to the compensation network and rectifier.

Figure 3b presents the voltage and current at the input of the primary circuit. The waveforms have an index, *i*, which stands for 1 in the case of DD1 resonator circuit and 2 in the case of DD2 resonator circuit. The rectangular input voltage waveform *V_Ai_* − *V_Bi_* is marked with the full red line, the first fundamental harmonic component of the rectangular voltage *u_i_* is marked with the dashed red line and the input current *i_Ti_* is marked with the blue line.

The primary circuits of the DD1 and DD2 are powered by a rectangular voltage. The transmitter and the receiver coils with their respected compensation capacitors form a band-pass filter. Therefore, near the resonant frequency, the rectangular voltage can be approximated using the first fundamental harmonic component of the voltages *u*_1_ and *u*_2_ [20]. The amplitude of the first fundamental harmonic component (*U*_1_ or *U*_2_) can be described with equation:

(1) $$U_{i}=\frac{4U_{DC}}{\pi }\sin\left(\frac{\pi }{2}\varphi_{i}\right)$$

where *U_DC_* is the voltage at the input of the inverter and φ*_i_* is the phase shift of the full-bridge inverter. The parameter *i* can be 1 or 2 for the *U*_1_ or *U*_2_ components. The voltage loop of the primary side of the DD1 circuit can be described using:

(2) $$U_{1}=R_{T1}I_{T1}+j\omega L_{T1}I_{T1}-j\frac{1}{\omega C_{T1}}I_{T1}-j\omega M_{1}I_{R1}$$

where *R_T_*_1_ is the DC resistance of the DD1 transmitter coil, *M*_1_ is the mutual inductance between the DD1 coils *L_T_*_1_ and *L_R_*_1_, and *I_T_*_1_ is the amplitude of the primary *i_T_*_1_ current and *I_R_*_1_ is the amplitude of the secondary *i_R_*_i_ current. The secondary side of the DD1 resonator circuit can be described with:

(3) $$j\omega M_{1}I_{T1}=R_{R1}I_{R1}+j\omega L_{R1}I_{R1}-j\frac{1}{\omega C_{R1}}I_{R1}+{R^{\prime }}_{L}I_{R}$$

where the load *R_L_* is reflected through the full bridge rectifier, and can be calculated using:

(4) $${R^{\prime }}_{L}=\frac{8}{\pi^{2}}R_{L}$$

Equation (4) is an approximated equation using the extended describing function (EDF), where only the first harmonic component of the voltage and the current is used. Load *R_L_*, connected to the secondary circuit using a rectifier, can be expressed using only resistance. The influence of the filtering capacitor *C_out_*_1_ on the primary and secondary circuits can therefore be neglected. The effect of the capacitor must be considered in the case of the expression of the output voltage transfer function.

The DD2 coil *L_T_*_2_ and compensation capacitor *C_T_*_2_ form a resonant circuit, powered by the sinusoidal voltage source *U*_2_ described with equation:

(5) $$U_{2}=R_{T2}I_{T2}+j\omega L_{T2}I_{T2}-j\frac{1}{\omega C_{T2}}I_{T2}$$

where *U*_2_ is the amplitude of the first fundamental harmonic component of *u*_2_, *R_T_*_2_ is the resistance of the DD2 transmitter coil, *I_T_*_2_ is the amplitude of the primary *i_T_*_2_ current, and *I_R_*_2_ is the amplitude of the secondary current *i_R_*_2_. The equation describing the primary side of the DD2 resonant circuit is similar to (2), although it does not include the effect of mutual inductance, due to the fact that current *I_R_*_2_ through *L_R_*_2_ on the secondary side is zero. The amplitude of the open loop voltage *U_M_* across the contacts of *L_R_*_2_ can be calculated as:

(6) $$U_{M}=j\omega M_{2}I_{T2}$$

where *M*_2_ is the mutual inductance between the DD2 coils *L_T_*_2_ and *L_R_*_2_. Current amplitude *I_T_*_2_ and open loop voltage amplitude *U_M_* are measured to calculate the mutual inductance. Mutual inductance *M*_2_ can then be calculated from (6) using:

(7) $$M_{2}=\frac{U_{M}}{2\pi f_{s}I_{T2}}$$

where *f_s_* is the operating frequency of the IPT system in the case that the transmitter and receiver pads are perfectly aligned, and only separated by the vertical distance *M*_1_ = *M*_2_. During the coupling coefficient measurement, the transmitter coil is unloaded. It is important to mention that the resonant frequency of the unloaded circuit should not be equal to the switching frequency of the inverter. In the resonance, the current is limited only by the coil wire resistance. Therefore, during the coupling coefficient measurement, the switching frequency should be increased to limit the sinusoidal current, by increasing the unloaded circuit impedance. Additionally, the first harmonic component of voltage *U*_2_ can be decreased by using smaller phase-shift *φ*_2_, thereby, additionally, lowering the current *I_T_*_2_.

The coupling coefficient between the DD2 coils can then be calculated using:

(8) $$k_{2}=\frac{M_{2}}{\sqrt{L_{T2}\cdot L_{R2}}}$$

The calculated mutual inductance and coupling coefficient *k_2_* and *M_2_* can then be used in the algorithms for the IPT system control optimisation, or in the algorithms for foreign object detection.

### 3.2. Mutual Inductance Measurement Using DD2 Coils

**Figure 4:**
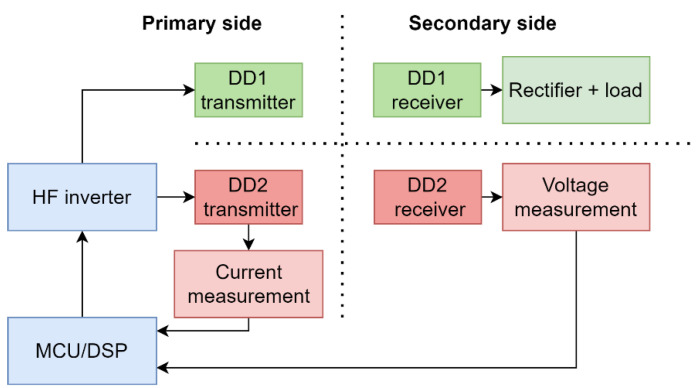
Block diagram of mutual inductance measurement.

When wireless power is transferred using the DD1 coils, the DD2 coils can be used to measure the mutual inductance. The receiver DD2 coil is disconnected from the compensation capacitor and rectifier and connected to the voltage measurement circuit. The structure of the circuit is the same as in Figure 3. The open-loop voltage *U_M_* across the contacts of the receiver DD coil is proportional to the mutual inductance between the transmitter and receiver coil and the current through the transmitter DD2 coil. The measurement can be represented by the block diagram in Figure 4.

The microcontroller measures the current through the transmitter DD2 coil using a current shunt resistor and current amplifier. This current induces voltage on the secondary side. The voltage measurement circuit on the secondary side measures the amplitude of the inducted voltage and transfers the value to the microcontroller. The voltage measurement circuit was produced using a high-impedance voltage divider and peak detector circuit using an operational amplifier. Amplitude data can be transferred to the microcontroller wirelessly. In the case of this paper, for the sake of simplicity, data were transferred via a wire. The microcontroller can calculate mutual inductance using Equation (7) and the coupling coefficient using Equation (8). Mutual inductance can be measured regardless of the DD1 transmitter coil. If the transmitter and receiver pads are aligned, *k*_1_ is the same as *k*_2,_ and there is no need for the *k*_1_ measurement.

## 4. Mutual Inductance Measurement Using 2DDq Coils

**Figure 5:**
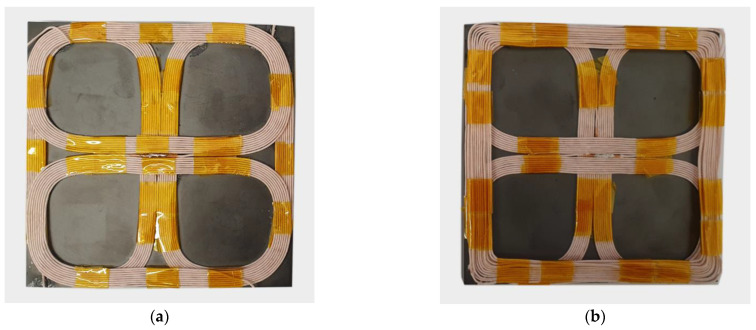
Double DD and 2DDq coil structure. (**a**) Double DD pad; (**b**) 2DDq pad.

On-line coupling coefficient measurement using double DD coils requires that one of the DD coils is disconnected from the load and connected to the voltage measurement circuit. This reduces the power transfer density by 50%. A different transmitter and receiver coil structure can be used as an alternative. This alternative pad consists of double DD coils with the addition of a quadrature q coil. This transfer pad structure can be named a 2DDq coil structure. Due to the nature of the DD coils, the coupling coefficient between the DD coils and the q coil is zero. Therefore, the transmitter DD1 and DD2 coils do not induce voltage in the transmitter q coil. The IPT system can use the DD1 and DD2 coils to transfer the power to the load and the q coil to induce voltage in the receiver pad q coil to measure the mutual inductance between the coils. The mutual inductance between the q coils is not the same as the mutual inductance between the DD1 coils and DD2 coils. Therefore, the measured value cannot be used directly in the control algorithms. The measured mutual inductance and coupling coefficient can be used for calculating the distance between the transfer pads. The transfer pads are presented in Figure 5. Figure 5a presents the double DD coil structure, presented in the previous chapter, and Figure 5b presents the proposed 2DDq coil structure, which would increase the power transfer capability of the IPT system.

If the transmitter and the receiver pads are misaligned, the DD transmitter coils can induce voltage in the receiver quadrature coil. This can affect the measured induced voltage on the receiver q coil. One of the possible solutions to this problem is to drive the transmitter q coil with a different frequency. An additional filter can be used between the receiver q coil and voltage measurement circuit to filter out the voltage induced due to the current in the DD1 and DD2 coils.

**Figure 6:**
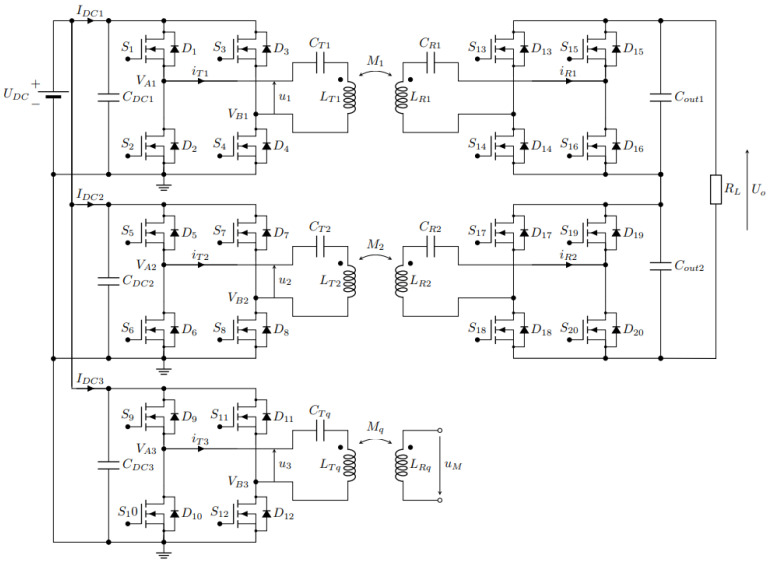
The system structure of IPT using a 2DDq coil structure.

The structure of the system is presented in Figure 6. The transmitter side requires three high-frequency inverters to drive the 2DDq coil structure. The DD1 and DD2 coils are driven using currents *i_T_*_1_ and *i_T_*_2_, respectively. The quadrature q coil, used for mutual inductance measurement, is driven with current *i_Tq_*. On the receiving side, two synchronous rectifiers are used to rectify the voltage induced in the receiver DD coils. The receiver q coil is connected to the voltage measurement circuit to measure the inducted voltage *U_M_*. The mutual inductance between the quadrature q coils can be calculated using:

(9) $$M_{q}=\frac{U_{M}}{2\pi f_{q}I_{Tq}}$$

where *U_M_* is the amplitude of the induced voltage, *I_Tq_* is the amplitude of the current *i_Tq_* through the transmitter quadrature q coil, and *f_q_* is the frequency of the voltage used to drive the q coil. The frequency *f_q_* should be above the resonant frequency of the circuit to limit the current of the unloaded circuit. Additionally, the first harmonic component of the excitation voltage can be lowered using phase-shifted modulation. The resonant frequency of the circuit can be calculated using:

(10) $$f_{r}=\frac{1}{2\pi \sqrt{L_{Tq}C_{T_{q}}}}$$

The resonant frequency *f_r_* is determined by the self-inductance *L_Tq_* of the transmitter coil and the series compensation capacitor with value *C_Tq_*. In order to limit the current through the unloaded coil, the circuit should operate above the resonant frequency.

The coupling coefficient between the transmitter and the receiver q coil can be calculated from the self-inductances of the q coils:

(11) $$k_{q}=\frac{M_{q}}{\sqrt{L_{Tq}\cdot L_{Rq}}}$$

The calculated mutual inductance *M_q_* is not the same as mutual inductance *M*_1_ and *M*_2_ between the DD coils. Therefore, the calculated value cannot be used directly in the control and optimisation algorithms.

## 5. Foreign Object Detection Method

The foreign object detection (FOD) system can be implemented using additional coils [10,18,21], cameras, radars, and ultrasonic sensors. Foreign objects can be also detected as a change in transfer efficiency or output voltage, or an increase in transmitter current. A detection algorithm identifies these changes and determines the foreign object.

When the FOD system detects a foreign object in the magnetic field, the IPT system is powered off to prevent injuries and power losses and notifies the user. The FOD in an IPT system with double DD coils can be implemented based on mutual inductance measurement. When a foreign metallic object is placed in the magnetic field of the active transmitter coil, the mutual inductance between the transmitter and receiver coil changes. If the inductance between the aligned coils decreases, this can be interpreted as a foreign object placed into the magnetic field. The advantage of the FOD based on mutual inductance measurement is simplicity—it does not require additional auxiliary coils, which increase system complexity and cost.

The FOD method using only mutual inductance change for detection has several disadvantages. One is that the system cannot distinguish between mutual inductance change due to a foreign object, or distance increase, or misalignment. In all cases, there is a change in the mutual inductance.

As described in [17], if both coils are active, the magnetic field radiated by the double DD coil interferes constructively and destructively, resulting in active regions with higher magnetic flux density and blind regions with no magnetic flux density. The active double DD coil has two active regions and two blind regions. If the foreign object is placed in a blind region, there is no magnetic field to induce voltage in the object. The influence of the object on the magnetic field cannot be measured, and the object cannot be detected. This means that, without special techniques, such as a rotating magnetic field, objects cannot be detected if they are located in blind regions. The advantage of this method is that it can detect objects without using additional coils that cannot contribute to wireless power transfer. After the FOD method, the coil, used for mutual inductance calculation, can be used in wireless power transfer. The system continues to transfer power with the double DD coil structure. The advantages of the double DD coil structure are presented in [17].

### Foreign Object Detection Using a Rotating Magnetic Field

**Figure 7:**
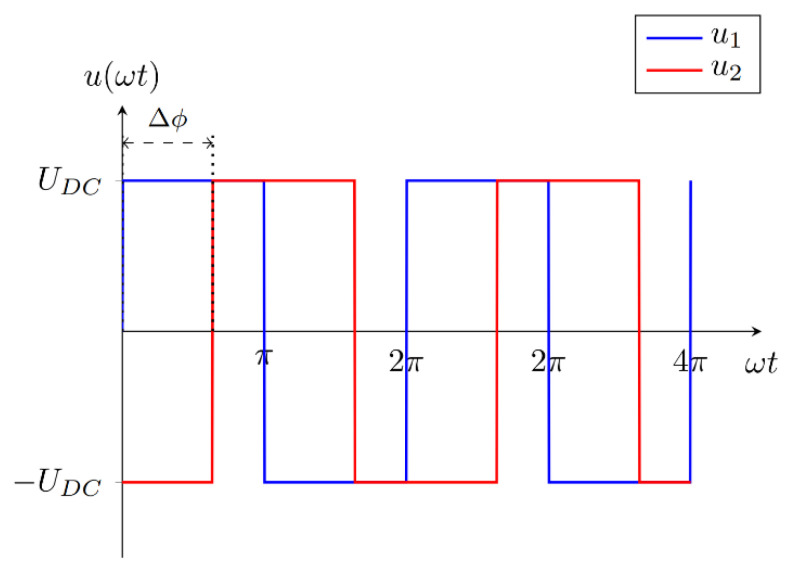
Phase-shifted high-frequency inverter square output voltage.

Double DD coils can also be used to generate a rotating magnetic field. This is the result of interference between the magnetic flux densities generated by currents in the DD1 and DD2 coils and the phase angles between them. The current in each of the DD transmitter coils is generated using the square output of the high frequency of the inverter. The DD1 transmitter coil is excited with the voltage denoted *u*_1,_ and the DD2 transmitter coil is excited with the voltage denoted with *u*_2_. The voltage *u*_1_ drives current *i_T_*_1_ and the voltage u_2_ drives current *i_T_*_2_. The phase angle Δ*ϕ* between the inverter voltages also reflects in the phase angle Δ*ϕ* between currents *i_T_*_1_ and *i_T_*_2_. The phase shifted voltage waveforms are presented in Figure 7.

**Figure 8:**
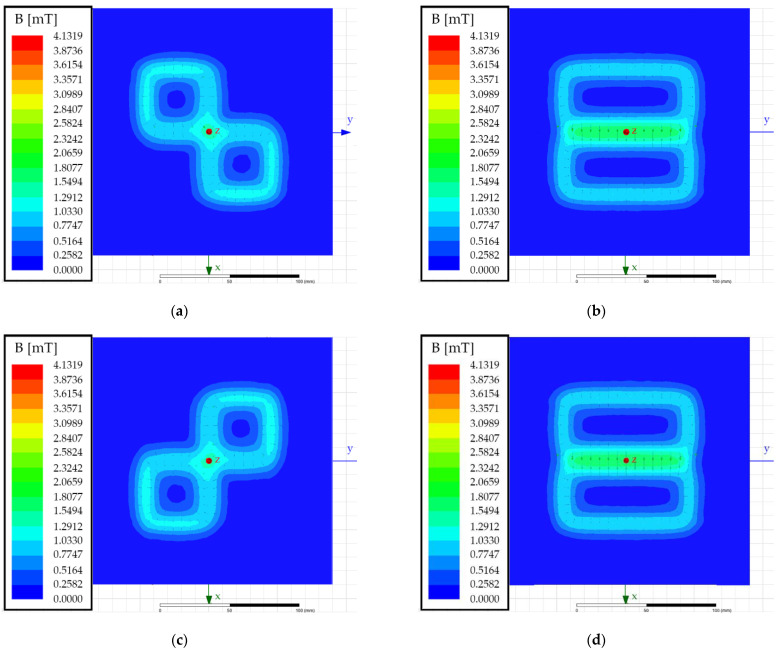
EM field simulations of a double DD transmitter pad in the *x*–*y* plane at *z* = 0 mm: (**a**) Phase angle 0°; (**b**) phase angle 90°; (**c**) phase angle 180°; (**d**) phase angle 270°.

Using Ansys Maxwell, a double DD transmitter pad can be modelled in 3D space and the magnetic flux density generated by the two phase-shifted currents can be simulated. The simulation results at four different phase angles in the *x*–*y* plane at *z* = 0 mm are presented in Figure 8. The DD1 and DD2 coils were both excited with a 1 A current. The simulation results were used to test and confirm the theory behind the behaviour of the double DD coil structure.

Figure 8a presents the magnetic flux density at phase angle 0°, Figure 8b presents the magnetic flux density at phase angle 90°, Figure 8c presents the magnetic flux density at phase angle 180°, and Figure 8d presents the magnetic flux density at phase angle 270°. At phase angles 0° and 180°, the double DD coils generate blind regions. As stated above, if the foreign object is placed in a blind region, it will not interact and transform the magnetic field. Therefore, change cannot be measured in the mutual inductance. Blind regions are formed in the shape of quadrants, which confirms that the rotating position of the blind and active regions can be used in the FOD algorithm.

From Figure 8, it can be observed that if the phase angle between currents is changed, the interference pattern of the radiated magnetic flux density also changes, and the blind and active regions rotate around the centre of the pad. The main magnetic flux path also rotates due to the phase angle between the primary DD1 and DD2 currents. The foreign objects that were previously in the blind spot and did not interfere with the magnetic field can now interact with the magnetic field and induce changes in mutual inductance between the transmitter and the receiver coil. If the foreign object is not present in the rotating magnetic field, the measured inductance does not change. The measured inductance value remains constant, regardless of the phase angle.

**Figure 9:**
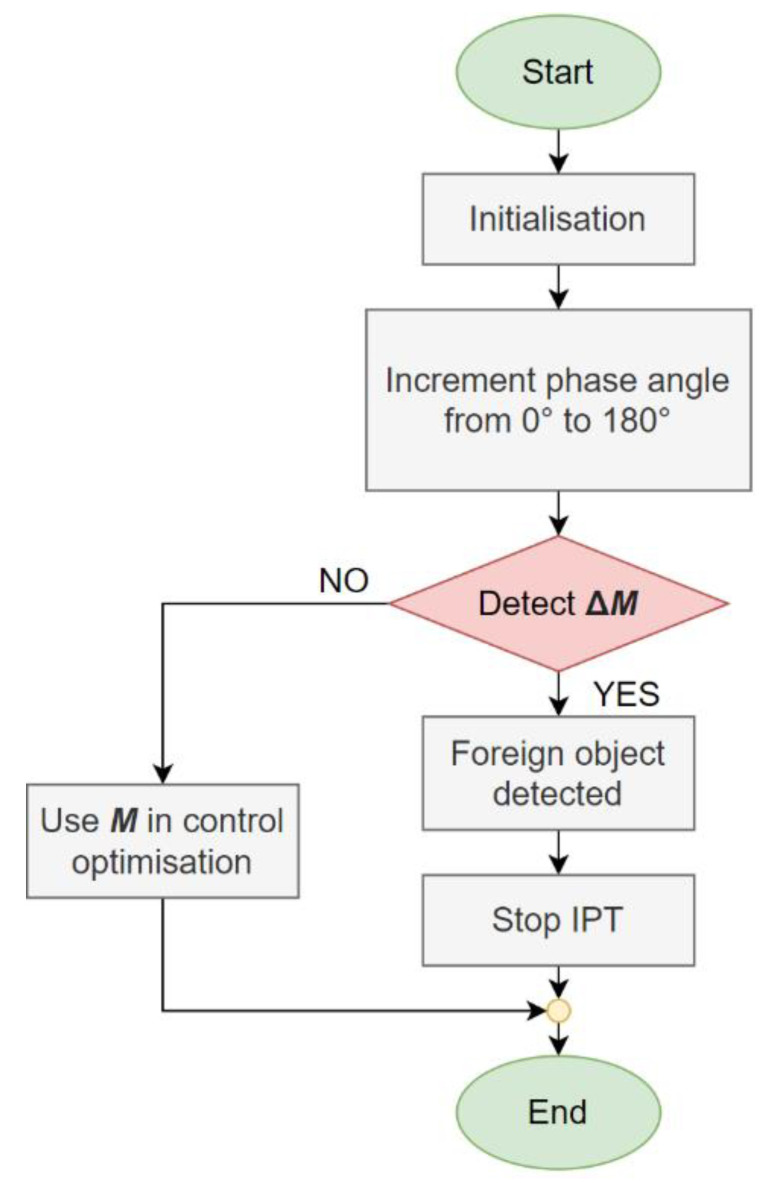
Foreign object detection algorithm.

The algorithm for foreign object detection is presented in Figure 9. It starts with the initialisation and measurement of the mutual inductance. The phase angle between the high-frequency inverter output voltage is incremented from 0° to 180° and back to 0°. At each increment, the mutual inductance is measured between the transmitter and the receiver coil. If a larger change in mutual inductance is detected, the foreign object is present in the field. The IPT system is turned off safely. If there is no larger mutual inductance change, and no foreign objects are present in between the transmission pads. The measured mutual inductance value can be used further in the IPT control optimisation.

The proposed FOD algorithm can then distinguish between inductance change due to the distance change between the transmitting and receiving pads and inductance change due to the foreign object in the field.

## 6. System Description

An experimental set bench was set up to evaluate the proposed simultaneous wireless power transfer and inductance measurement. The set bench consisted of two parts:

- A 3D computer-controlled positioning rig;
- An IPT system with the required measuring circuits.

The double DD transfer pads were mounted on the movable platforms of the 3D positioning measurement rig. The rig enabled precise positioning of the DD coils in a 3D space. Misalignment and the distance between the transmitter and receiver coils were controlled by a computer application. The computer application also interacted with the IPT system. Positioning the coils in the 3D space enabled the measurement of the mutual inductance and coupling coefficient between the transmitter and receiver pads at different distances and at different misalignments.

**Figure 10:**
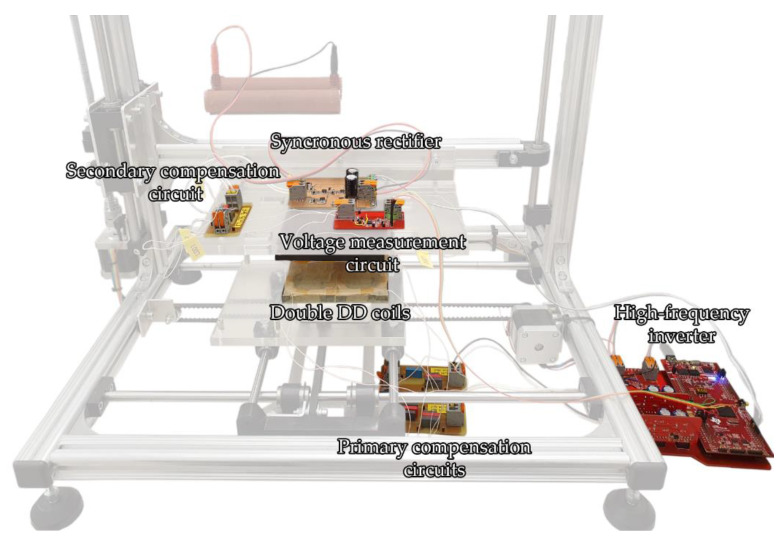
The experimental set workbench of the IPT system with the double DD coil structure and voltage measurement circuit.

The primary side of the IPT system consisted of two full-bridge inverters, which were connected to two series resonant compensation circuits for the DD1 and DD2 coils. On the secondary side, only the DD1 coil was connected directly to the secondary side compensation circuit, synchronous rectifier, and load. The DD2 coil was used for the measurement of the coupling coefficient. It was connected to a high-impedance voltage measurement circuit. The voltage measurement circuit was used to measure the open-loop voltage induced in the DD2 coil because of the primary current through the TX DD2 circuit. The primary TX DD2 circuit behaved as an unloaded wireless transfer circuit and did not interact with the DD1 RX coil on the secondary side. The system is presented in Figure 10. The coils were mounted on the 3D positioning mechanism. The highlighted components and circuits are the main parts of the system, necessary for its operation, which include the high frequency inverter, the primary and the secondary compensation circuits, the synchronous rectifier, and the voltage measurement circuit.

The operating points of the experimental set workbench are presented in Table 1.

### 6.1. High-Frequency Inverter

The high-frequency inverter was rated for voltage up to 50 V. It consisted of two full-bridge MOSFET inverters and two isolated transistor driving circuits. The transistor full-bridges were connected to the output, which was connected to three primary resonant circuits. The first full-bridge inverter was used to drive the DD1 TX coil with *u_T_*_1_, and the second full-bridge inverter was used to drive the DD2 TX coil with *u_T_*_2_. The current *I_T_*_1_ through the DD1 primary circuit and current *I_T_*_2_ through the DD2 primary circuit were measured using a current shunt resistor and a current sense amplifier, which measured and amplified the voltage across the current shunt. The high-frequency inverter was connected to a PC using a USB cable. The PC enabled inverter control and data logging.

### 6.2. Primary and Secondary Resonant Circuits

The primary circuit consisted of two primary series resonant capacitors *C_T_*_1_ and *C_T_*_2_, used to compensate and resonate with inductances *L_T_*_1_ and *L_T_*_2_ of the DD1 and DD2 coils. The secondary series resonant circuit with capacitance *C_R_*_1_ resonated with the inductance *L_R_*_1_ of the DD1 receiver coil. The DD2 receiver coil was connected to the voltage measurement circuit, and therefore did not need a compensation capacitor. When the mutual inductance measurement was performed, the DD2 receiver coil could be connected to the compensator circuit and rectifier.

### 6.3. Synchronous Rectifier

The synchronous rectifier was designed to rectify voltages up to 50 V DC. The rectifier consisted of four MOSFETs as a replacement for the diodes, to eliminate diode voltage drop losses. The MOSFETs were driven using four synchronous rectifier gate drivers that drive MOSFETs based on AC input voltage. The rectifier circuit also included a current shunt and a current amplifier for the DC current measurement, and a DC output voltage measurement circuit using a voltage divider and amplifier.

### 6.4. Voltage Measurement Circuit

This circuit was connected directly to the receiver DD2 coil with inductance *L_R_*_2_. The circuit had high input impedance. An open circuit voltage is required to calculate mutual inductance. The measurement circuit was designed to measure AC voltage up to 50 V. The circuit included a peak detection circuit, which detects the peak value of the AC voltage, which is then used in the mutual inductance calculation in combination with the measured current from the transmitter side.

## 7. Measurement Results

This chapter presents the measurement results of the coupling coefficient measurements using the double DD coil structure and the 2DDq coil structure. Foreign object detection was performed only on the DD coil structure, as the 2DDq coil structure does not enable measurement using the rotation of a directional magnetic field.

### 7.1. On-Line Coupling Coefficient Measurement Results Using the DD Coil

This section presents the results of the coupling coefficient measurement on the IPT system and foreign object detection. The DD1 coil in the double DD coil structure was used for power transfer, and the DD2 coil in the double DD coil was used for the mutual inductance measurement.

The first coupling coefficient measurement was measured when the coils were perfectly aligned. The distance between the transfer pads was varied from 15.3 mm to 55.3 mm on the *z*-axis at 1 mm increments. At each point, the primary current *I_T_*_2_ and open circuit voltage *U_M_* were measured and used for the on-line calculation of the mutual inductance and coupling coefficient. The measurement data for a couple of measurement points are presented in Table 2. The Table includes the distance between the pads *z*, the inductance of the DD2 transmitter and the receiver coils *L_T_*_2_ and *L_R_*_2_, the transmitter excitation current *I_T_*_2_, and the open-circuit-induced voltage *U_M_*.

**Figure 11:**
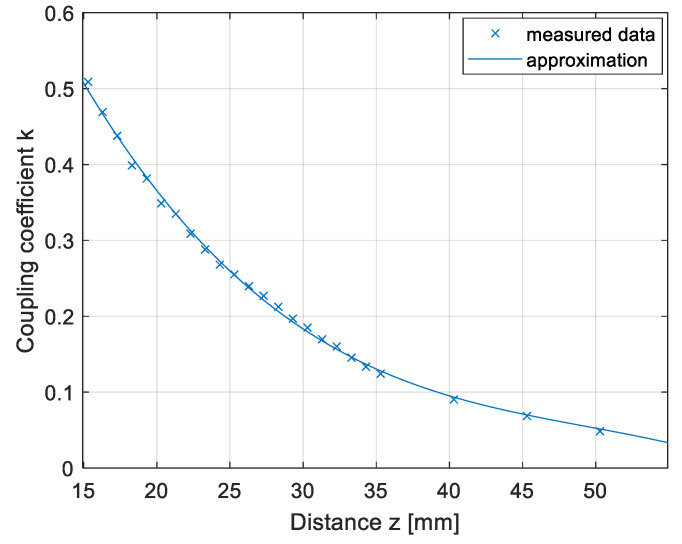
Coupling coefficient measurement in the *z* direction.

The results are presented in Figure 11. The coupling coefficient calculated from the measurements is marked with crosses, and the approximated data are marked with a full line. If the transfer pads were not misaligned horizontally, the coupling coefficient of the DD1 coil and DD2 coil were the same.

**Figure 12:**
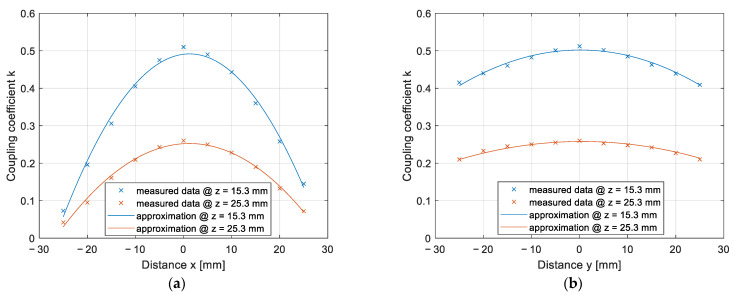
Coupling coefficient measurement on the *x*–*y* plane: (**a**) In the *x* direction; (**b**) in the *y* direction.

The results of the coupling coefficient change due to the horizontal *x*–*y* misalignment are presented in Figure 12a,b. The measurements were performed at two different vertical distances between the pads: 15.3 mm and 25.3 mm. The measurements at 15.3 mm are marked with the blue curve and measurements at 25.3 mm are marked with the orange curve. Figure 12a presents the results of misalignment along the *x*-axis, and Figure 12b presents the results of misalignment along the *y*-axis. The coupling coefficients calculated from the measurements are marked with crosses, and the approximated data are marked with a full line.

The misalignment of DD2 coils along the *x*-axis had a greater impact on the coupling coefficient and, therefore, the transfer efficiency, compared to the drop in coupling coefficient along the *y*-axis direction. On the other hand, the misalignment across the *y*-axis had a greater impact on the coupling coefficient of the DD1 coil, compared to the misalignment along the *x*-axis.

### 7.2. On-Line Coupling Coefficient Measurement Results Using the 2DDq Coil

This section presents the results of the coupling coefficient measurements on the IPT system using the 2DDq transmitter and receiver pad structure. The DD1 and DD2 coils were used to transfer power to the load on the receiving side, and the transmitter quadrature q coil was used to measure the mutual inductance between the transmitter and the receiver coil, the q coil. The mutual inductance between the q coils was not the same as the mutual inductance between the transmitter and the receiver double DD coils. Therefore, the measured mutual inductance can be used to calculate the distance or misalignment between the pads. The measured value cannot be used directly in control optimisation. The coupling coefficient was calculated using measurements of the transmitter *L_Tq_*, coil current *I_Tq_* and the induced open loop voltage *U_M_*, measured on the receiver coil *L_Rq_*. A couple of the measurement points are presented in Table 3. The coupling coefficient was calculated in the same way as in the case of the DD2 coils. The only difference was in the values of the q measurement coils.

**Figure 13:**
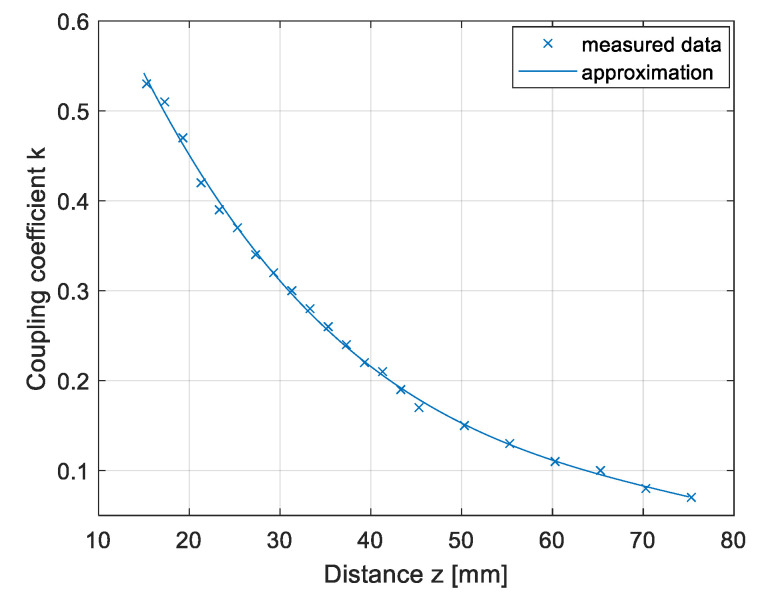
Coupling coefficient measurement in the *z* direction.

The results of the coupling coefficient measurement in the *z* direction are presented in Figure 13. The coupling coefficient calculated from the measurements is marked with crosses and the approximated data are marked with the full line. The distance between the transmitter and receiver pad was varied from 15.3 mm to 75.3 mm in 2 mm increments. The largest coupling coefficient between the transmitter and the receiver q coil was 0.53, which was larger than the coupling coefficient between the DD1 and DD2 coils.

The coupling coefficient can be approximated using the equation:

(12) $$k_{3}\left(z\right)=6.64\cdot 10^{-9}z^{4}-2.99\cdot 10^{-6}z^{3}+4.56\cdot 10^{-4}z^{2}-3.16\cdot 10^{-2}z+0.92$$

The transmitter and the receiver pads are aligned when the currents *I_T_*_1_ and *I_T_*_2_ are the same.

**Figure 14:**
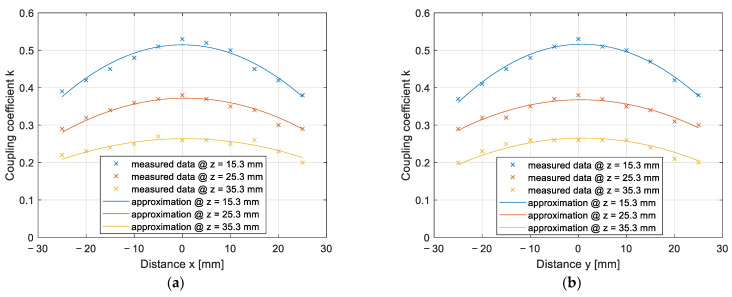
Coupling coefficient measurement on the *x*–*y* plane: (**a**) In the *x* direction; (**b**) in the *y* direction.

The results of the impact of horizontal misalignment in the *x* and *y* axes on the coupling coefficient are presented in Figure 14. The coupling coefficients calculated from the measurements are marked with crosses, and the approximated data are marked with a full line. The measurements were performed at three different *z* distances. Figure 14a presents the results of the coupling coefficient measurement on the *x* axis, when *y* = 0 mm, and Figure 14b presents the results of the coupling coefficient measurement on the *y* axis, when *x* = 0 mm. In both Figures, the blue line represents the coupling coefficient measurement at *z* = 15.3 mm, the red line represents measurements at *z* = 25.3 mm, and the yellow line represents measurements at *z* = 35.3 mm. Compared to the measurements with the DD coils, presented in Figure 11, the q coils have a symmetrical misalignment tolerance.

### 7.3. Foreign Object Detection Results

**Figure 15:**
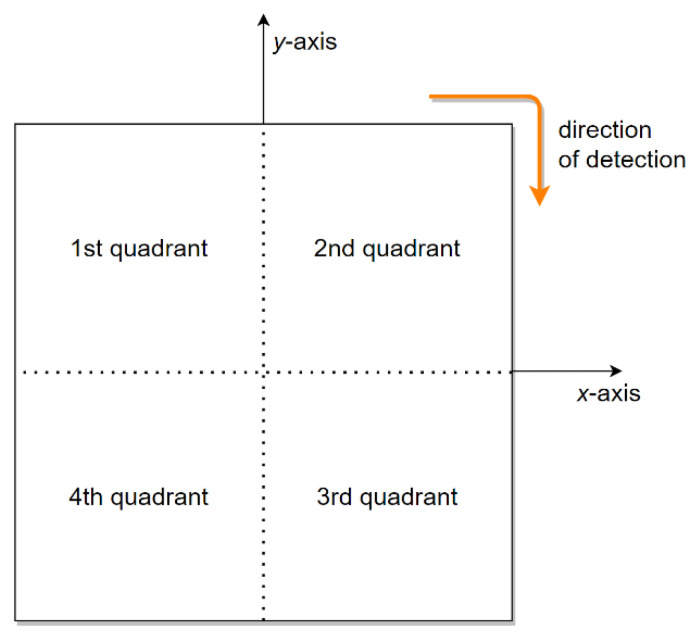
The double DD coil structure divided into four quadrants.

For the sake of better representation, the double DD coil structure can be divided into four quadrants, as presented in Figure 15. The direction of the rotating magnetic field is indicated with the orange arrow, from the 1st to the 2nd to the 3rd and, lastly, to the 4th quadrant. When the phase angle between the input voltages is 0°, the 1st and 3rd quadrants are linked magnetically, and the 2nd and 4th quadrants are blind regions. On the other hand, when the phase angle between the input voltages is changed to 180°, the 2nd and 4th quadrants are linked magnetically, and the blind regions are in the 1st and 3rd quadrants.

A foreign object can be placed into one or multiple quadrants. Using the measured mutual inductance and coupling coefficients, the proposed method can determine whether the change in mutual inductance is the result of a foreign object, or simply to the change in distance between the transfer pads. To rotate the magnetic field correctly, the currents through DD1 and DD2 must be the same. In the following results, the transmitter and receiver pads were 15.3 mm apart.

**Figure 16:**
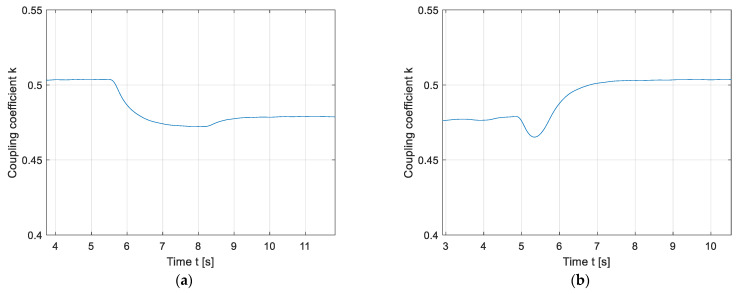
Stationary magnetic field foreign object detection at phase angle 0°: (**a**) Placement of the object in the 1st quadrant; (**b**) movement of the object from the 1st to the 2nd quadrant.

**Figure 17:**
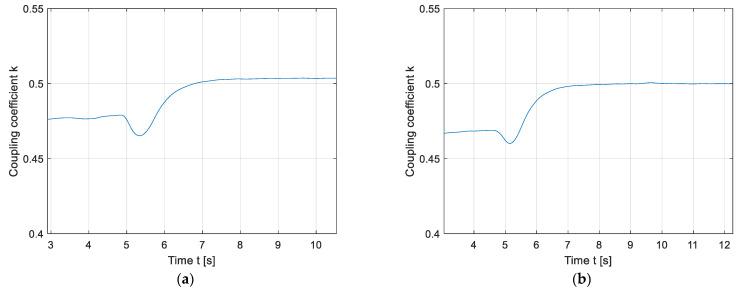
Stationary magnetic field foreign object detection at phase angle 180°: (**a**) Placement of the object in the 2nd quadrant; (**b**) movement of the object from the 2nd to the 1st quadrant.

Firstly, the results of the static magnetic field are presented in Figure 16 and Figure 17. Figure 16 presents the impact of a foreign object on the coupling coefficient when the phase angle between driving voltages was 0°. If the object was placed in the 1st quadrant, the coupling coefficient decreased, which is evident from Figure 16a. Figure 16b presents the coupling coefficient change when the object was moved from the 1st quadrant to the second quadrant. At phase angle 0°, the 2nd quadrant is a blind region, which cannot sense foreign objects. This results in a higher coupling coefficient measurement, which is the same as in the case when the foreign object is not present between the transmitter and the receiver pad. The same coupling coefficient fluctuations was observed when the object was placed in the 3rd quadrant and then moved to the 4th quadrant. The coupling coefficient between the transmitter DD2 coil and the receiver DD2 coil was measured and calculated in the same way as is presented in Table 2.

Figure 17 presents the impact of a foreign object on the coupling coefficient when the phase angle between driving voltages was 180°. Figure 17a presents the reduction in the coupling coefficient when the foreign object was placed in the 2nd quadrant. When the object was moved from the 2nd quadrant to the 1st quadrant the coupling coefficient increased, which can be seen in Figure 17b. The 1st quadrant is a blind area, and therefore does not result in a change in the coupling coefficient value. An object in the 1st quadrant cannot be detected. The same coupling coefficient fluctuation as seen in Figure 17 was observed if the object was placed in the 4th quadrant and then moved to the 3rd quadrant.

The results in Figure 16 and Figure 17 showcase the shortcomings and limitations of the coupling coefficient measurements in a static magnetic field. If the object was not located in an active region, it could not be detected. Additionally, the decrease or increase in mutual inductance can be attributed to the change in distance between the transmitter and the receiver pad.

**Figure 18:**
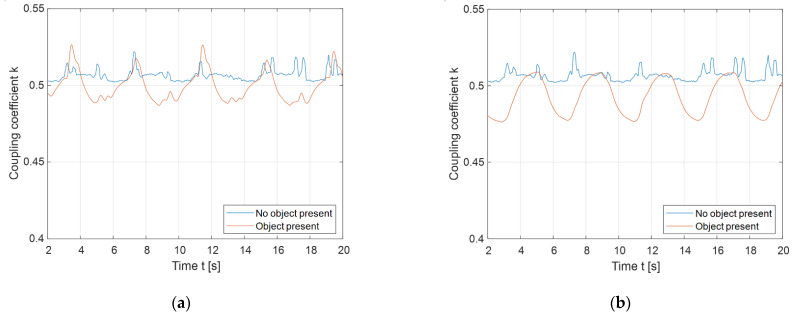
Rotating magnetic field foreign object detection: (**a**) Placement of the object in the 1st quadrant; (**b**) placement of the object in the 2nd quadrant.

On the other hand, the detection of foreign objects can be improved by utilising a rotating magnetic field, by varying the phase angle between the output voltages of the high-frequency inverter. The phase angle between voltages can be varied from 0° to just 180° because of the double DD coil symmetry. The results of the foreign object detection using a rotating magnetic field are presented in Figure 18. Figure 18a presents the change in the measured coupling coefficient when the object was placed in the 1st or 3rd quadrants. The blue line presents the baseline, when there was no object placed in the rotating magnetic field. The orange line presents the periodic fluctuation of the measured coupling coefficient when the foreign object was present. The object caused the orange line to drop below the blue line, which clearly indicates the presence of the object.

Similar fluctuations in the measured coupling coefficient can be observed in Figure 18b, when the object was placed in the 2nd or 4th quadrants. The blue line also represents the baseline without the foreign object, and the orange line also presents the coupling coefficient fluctuations because of the interaction between the rotating magnetic field and the foreign object. When the object was detected, the value of the coupling coefficient decreased. There is a clear difference in the change in the coupling coefficient due to the presence of the foreign object in the static and rotating fields. In the rotating field, the presence of the foreign object cannot be confused with the change in distance between the transmitter and the receiver pad.

**Figure 19:**
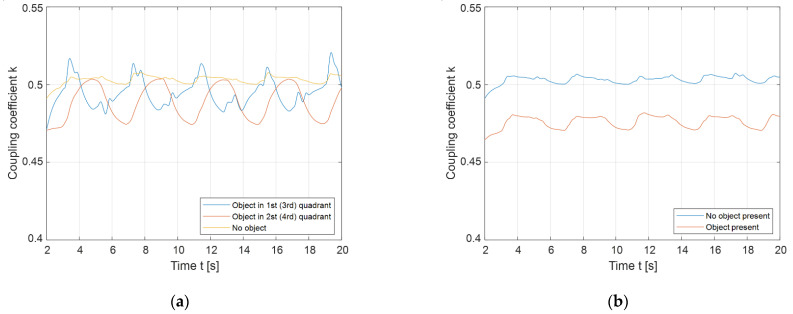
Rotating magnetic field foreign object detection: (**a**) Comparison of the object detected in the 1st quadrant to the object detected in the 2nd quadrant; (**b**) object detection in the 1st and 2nd quadrants.

Figure 19a presents the difference between the coupling coefficient waveforms when the object was placed in the 1st (3rd) quadrant (blue line) or 2nd (4th) quadrant (orange line). The yellow line represents the coupling coefficient baseline when no object was present. The object in the 1st quadrant is 90° ahead of the object in the 2nd quadrant. This results in the blue signal preceding the orange signal by approximately 90°. Based on the current phase angle, the FOD can determine whether an object is placed in the 1st (3rd) or 2nd (4th) quadrants.

Figure 19b presents the coupling coefficient change when the object was presented in both the 1st and 2nd quadrants at the same time. When the object was present, the coupling coefficient represented with the orange line decreased under the baseline. In this case, it is more difficult to determine whether the reduction in the coupling coefficient was due to the foreign object, or because the distance between transfer pads was increased. In this case, the results of FOD using the rotating magnetic field were little different from the results of FOD using the static magnetic, non-rotating magnetic field.

## 8. Discussion

The proposed method enables the measurement of mutual inductance and coupling coefficients during wireless power transfer. Because one of the coils must be open-loop, only one of the DD coils transfers power and the power density is halved. The measurement coil is disconnected from the resonant circuit and connected to the voltage measurement circuit. If the transfer pads are aligned horizontally, the measured mutual inductance is the same as the mutual inductance between the power transmitter and the receiver coils. If there is misalignment due to the nature of the DD coils, the mutual inductances are different. To determine the mutual inductance between two misaligned transfer pads, both DD1 and DD2 mutual inductances must be measured, which requires two voltage measurement circuits instead of just one. The power transfer using misaligned coils is usually suboptimal, so the best way to optimise the efficiency of a wireless power transfer is to detect the misalignment and notify the user of the charger.

A different IPT coil structure, using a different driving circuit, was used to increase the transfer power. The proposed structure consists of two transmitter DD coils and one quadrature q coil. Both DD coils are used to transfer power, and the q coil is used to measure the mutual inductance. The measured mutual inductance between the q coils is different from the mutual inductance between the transmitter and the receiver DD coils. This is mainly because DD coils and q coils differ in type, number of turns, and size. Therefore, the measured value cannot be used directly in control optimisation. Additionally, the DD coil can induce voltage in the q coil if the transfer pads are misaligned horizontally. Consequently, the transmitter q coil is driven with a different frequency, which enables the filtering out of the voltage induced with the DD coils.

A change in the mutual inductance during charging can also indicate the presence of a foreign metallic object between the transmitter and the receiver pad. Instead of a static directional magnetic field, the double DD coil structure enables the use of a rotating directional magnetic field. The magnetic field can be rotated by changing the phase shift between the high-frequency inverter output voltages. The directed field rotates around the centre of the transfer pad. If the mutual inductance remains the same, the transfer coils are aligned perfectly, and there is no foreign object between pads. If the mutual inductance changes periodically, there is a foreign object or misalignment between the transmitter and the receiver pads. If the pads are aligned and without additional metallic abstraction, the mutual inductance remains constant, because the magnetic field is uniform and unabstracted. This FOD method can easily determine whether the change in the mutual inductance is due to the change in distance between the transmitter and the receiver coil, or whether there is a foreign object located in the field. When the phase angle between the excitation currents does not change, there is almost no way to determine whether the change in mutual inductance is due to the presence of a foreign object, which can be seen in Figure 16 and Figure 17. When the phase angle changes, it can clearly be determined that the object is present, as seen in Figure 18.

The proposed FOD can be used in all systems using a double DD coil structure, without the additional cost of including special foreign detection coils. Additionally, the double DD coil structure enables higher power density, reduces stress on the circuit components, and has the symmetrical horizontal misalignment tolerance described further in [17]. The proposed FOD method can be used at the start of the wireless power transfer, and is proposed and tested for static IPT systems. The FOD method would need to be modified and synchronised with the control algorithm, running in real time to work on dynamic IPT systems, and will be the subject of forthcoming research.

The main drawbacks of the proposed method are presented in Figure 19. When the object is presented in quadrants that are next to each other, such as the 1st and 2nd coil quadrants, the coil cannot determine whether the object is present or whether the distance between the transmitter and receiver coil has changed. During the test, two objects of the same shape and size were placed in the magnetic field. The objects could still be detected if there was a difference in sizes between the objects, due to the larger change in the mutual inductance. An additional limitation of the method is that the transmitter coil and the receiver coil need to be aligned horizontally for the method to work properly. At larger misalignments, the measured mutual inductance changes due to the different coupling coefficients, *k*_1_ and *k*_2_, between the two DD1 and two DD2 coils.

## 9. Conclusions

This paper presents an on-line foreign object detection system using a double DD coil pad structure. If the transmitter and receiver double DD coil structure are not rotated to each other, the system can be used as two magnetically uncoupled circuits. This allows one DD coil pair to be used for power transfer, and the other pair for mutual inductance measurement. However, this also reduces the power transfer efficiency by half. As a solution to this problem, a different coil topology, called 2DDq coil topology, was also used. This topology can be used to detect changes in alignment and to determine the distance between the transmitter and the receiver pad.

In this study, the coupling coefficient was measured using the open-loop receiver voltage measurement method. The value of mutual inductance can be used to determine the distance between horizontally aligned transfer pads, or in control optimisation, which was not within the scope of this paper. The change in coupling coefficient can indicate two things. Firstly, it can indicate a change in distance between the transfer pads. Secondly, it can indicate the presence of a foreign object between the pads. By rotating the magnetic field using the phase shifted excitation current, the algorithm can differentiate between these two causes, and can determine the cause of impact on the coupling coefficient change. This enables FOD without the requirement of additional coils that cannot be used for wireless power transfer. In cases in which no object is detected, the coils used in the mutual inductance calculation can be used for wireless power transfer.

## Figures and Tables

**Table 1:** IPT system parameters.

Parameter	Value
Input voltage *U_DC_*	15 V
Input current *I_DC_*	2 A
Operating frequency *f_s_*	87 kHz
Inductance *L_T_*_1_	45 μH
Inductance *L_T_*_2_	41.6 μH
Inductance *L_R_*_1_	45.1 μH
Inductance *L_R_*_2_	41.8 μH
Capacitance *C_T_*_1_	76.8 nF
Capacitance *C_T_*_2_	84.5 nF
Capacitance *C_R_*_1_	76.9 nF
Resistance *R_L_*	10.7 Ω

**Table 2:** The coupling coefficient measurement between the DD2 coils in the *z* direction.

*z* (mm)	*L_T_*_2_ (μH)	*L_R_*_2_ (μH)	*I_T_*_2_ (A)	*U_M_* (V)	*k*
15.3	41.6	41.8	1.67	19.56	0.51
16.3	41.6	41.8	1.86	20.00	0.47
17.3	41.6	41.8	2.03	20.42	0.44
18.3	41.6	41.8	2.28	20.83	0.40
19.3	41.6	41.8	2.35	20.54	0.38
20.3	41.6	41.8	2.30	18.42	0.35
21.3	41.6	41.8	2.16	16.61	0.33
22.3	41.6	41.8	2.11	14.95	0.31
23.3	41.6	41.8	2.01	13.30	0.29
…	…	…	…	…	…
55.3	41.6	41.8	0.79	0.63	0.03

**Table 3:** The coupling coefficient measurement between the 2DDq coils in the *z* direction.

*z* (mm)	*L_Tq_* (μH)	*L_R_*_q_ (μH)	*I_Tq_* (A)	*U_M_* (V)	*k*
15.3	43	43.1	0.76	26.39	0.53
17.3	43	43.1	0.92	31.19	0.51
19.3	43	43.1	1.24	38.39	0.47
21.3	43	43.1	1.84	51.22	0.42
23.3	43	43.1	2.48	63.99	0.39
25.3	43	43.1	2.16	52.79	0.37
27.3	43	43.1	1.76	39.99	0.34
29.3	43	43.1	1.60	33.59	0.32
31.3	43	43.1	1.44	27.99	0.30
…	…	…	…	…	…
75.30	43	43.1	1.08	4.80	0.07

## Data Availability

Data are contained within the article at hand.
